# *Taenia solium* Infections in a Rural Area of Eastern Zambia-A Community Based Study

**DOI:** 10.1371/journal.pntd.0001594

**Published:** 2012-03-27

**Authors:** Kabemba E. Mwape, Isaac K. Phiri, Nicolas Praet, John B. Muma, Gideon Zulu, Peter Van den Bossche, Reginald de Deken, Niko Speybroeck, Pierre Dorny, Sarah Gabriël

**Affiliations:** 1 Department of Clinical Studies, School of Veterinary Medicine, University of Zambia, Lusaka, Zambia; 2 Department of Biomedical Sciences, Institute of Tropical Medicine, Antwerp, Belgium; 3 Department of Disease Control, School of Veterinary Medicine, University of Zambia, Lusaka, Zambia; 4 Petauke District Hospital, Petauke, Zambia; 5 Institute of Health and Society, Université Catholique de Louvain, Brussels, Belgium; 6 Laboratory of Parasitology, Faculty of Veterinary Medicine, Ghent University, Ghent, Belgium; University of Melbourne, Australia

## Abstract

**Background:**

*Taenia solium* taeniosis/cysticercosis is a parasitic infection occurring in many developing countries. Data on the status of human infections in Zambia is largely lacking. We conducted a community-based study in Eastern Zambia to determine the prevalence of human taeniosis and cysticercosis in a rural community.

**Methods and Findings:**

Stool and serum samples were collected from willing participants. Geographical references of the participants' households were determined and household questionnaires administered. Taeniosis was diagnosed in stool samples by coprology and by the polyclonal antibody-based copro-antigen enzyme-linked immunosorbent assay (copro-Ag ELISA), while cysticercosis was diagnosed in serum by the B158/B60 monoclonal antibody-based antigen ELISA (sero-Ag ELISA). Identification of the collected tapeworm after niclosamide treatment and purgation was done using polymerase chain reaction-restriction fragment length polymorphism (PCR-RFLP). A total of 255 households from 20 villages participated in the study, 718 stool and 708 serum samples were collected and examined. Forty-five faecal samples (6.3%) were found positive for taeniosis on copro-Ag ELISA while circulating cysticercus antigen was detected in 5.8% (41/708) individuals. The tapeworm recovered from one of the cases was confirmed to be *T. solium* on PCR-RFLP. Seropositivity (cysticercosis) was significantly positively related to age (*p* = 0.00) and to copro-Ag positivity (taeniosis) (*p* = 0.03) but not to gender. Change point analysis revealed that the frequency of cysticercus antigens increased significantly in individuals above the age of 30. Copro-Ag positivity was not related to age or gender. The following risk factors were noted to be present in the study community: free-range pig husbandry system and poor sanitation with 47.8% of the households visited lacking latrines.

**Conclusions:**

This study has recorded high taeniosis and cysticercosis prevalences and identified the need for further studies on transmission dynamics and impact of the disease on the local people.

## Author Summary

*Taenia solium* taeniosis/cysticercosis is a zoonotic infection endemic in many developing countries, with humans as the definitive host (taeniosis) and pigs and humans as the intermediate hosts (cysticercosis). When humans act as the intermediate host, the result can be neurocysticercosis, which is associated with acquired epilepsy, considerable morbidity and even mortality. In Africa, most studies have been carried out in pigs with little or no data in humans available. In this human study, conducted in a rural community in Eastern Zambia, prevalences for taeniosis and cysticercosis were determined at 6.3% and 5.8% respectively, indicating the hyperendemicity of the area. Cysticercosis infection was strongly related with age, with a significant increase in prevalence occurring in individuals from the age of 30 onward. A collected tapeworm was confirmed to be *T. solium*. Risk factors associated with the transmission and maintenance of the parasite such as free roaming pigs, households without latrines, backyard slaughter of pigs without inspection and consumption of undercooked pork were also present. The findings of this work have identified the need for further research in the transmission dynamics and the burden that this infection has on the resources of poor local people.

## Introduction

*Taenia solium* taeniosis/cysticercosis is a neglected parasitic zoonosis, affecting mostly developing countries [1]. Its occurrence is strongly associated with poverty, poor hygiene and sanitation, free-range pig management and lack of meat inspection [2]–[4]. Adult intestinal tapeworm infection (taeniosis) in man, who is the sole natural definitive host [5], is acquired by eating undercooked infected pork. Infective eggs are shed (with proglottids) via the stool and contaminate the environment. Pigs are the intermediate host and are infected by ingestion of these infective eggs (or proglottids), which develop into cysticerci (porcine cysticercosis). Humans can act as intermediate hosts as well; cysts can develop subcutaneously, intramuscularly, but often in the central nervous system causing neurocysticercosis (NCC). NCC has been described as the most frequently reported helminthic infection of the central nervous system [6] and is a major cause of acquired epilepsy in cysticercosis endemic regions associated with considerable morbidity [7].

In the last decade, many studies have been carried out in sub-Saharan Africa to determine the presence/absence of *T. solium*. Until now, most studies have been carried out on porcine cysticercosis reporting endemicity in countries like Tanzania, Zambia, Mozambique and Kenya [8]–[12]. Prevalence of human cysticercosis, which has been less studied, ranges from 7.4% in South Africa to 20.5% in Mozambique (both based on specific antibody detection) and 21.6% in the Democratic Republic of Congo (based on circulating antigen detection) [13]–[16]. In studies conducted in Kenya, taeniosis prevalences were estimated from 4 to 10% [17]. These data emphasize the need for more studies in humans to gather information on the epidemiology of the parasite and to estimate the burden on affected communities.

In Zambia, pig keeping and pork consumption have increased significantly during the past decade with Eastern and Southern provinces accounting for a greater part of this increase [4]. Pigs are mostly kept by smallholder producers, under free-range management. Several studies carried out in Zambia have indicated high prevalences of porcine cysticercosis. A study at a slaughter slab in Lusaka receiving village pigs, indicated a prevalence of up to 64.2% [18] while field studies in Southern and Eastern province estimated sero-prevalences between 10.8–20.8% and 9.3–23.3% respectively [19], [20]. These studies clearly showed that *T. solium* was present in rural areas of Zambia. However, except for preliminary unpublished data, no information was available on human cysticercosis/taeniosis.

The main objective of the current study was to determine the prevalence of human taeniosis and cysticercosis in a rural community of Petauke district in the Eastern province of Zambia.

## Materials and Methods

### Ethical statement

Ethical clearance (approval) for both human and animal parts of the study was obtained from the University of Zambia Biomedical Research Ethics Committee (IRB0001131). For the human part of the study, further approval was sought from the Ministry of Health of Zambia and also from the local District health authorities. A meeting was held with the community leaders (headmen) to explain the purpose of the study and request their permission to conduct the study in their area. Finally consent was also sought from the individual subjects to take part in the study. Subjects were not forced to participate and participation was requested of individuals of all ages after written informed consent. For individuals below the age of 16, permission was sought from their parents or guardians by way of written informed consent. All participants found positive for taeniosis and other helminths were provided with treatment, namely niclosamide and mebendazole respectively. Those positive for cysticercosis were referred to the District hospital for follow-up and the recommended standard of care provided to them if required.

Collection of reference samples from pigs at a local abattoir was carried out by professional veterinarians adhering to the Zambian regulations and guidelines on animal husbandry.

### Study area and population

**Figure 1:**
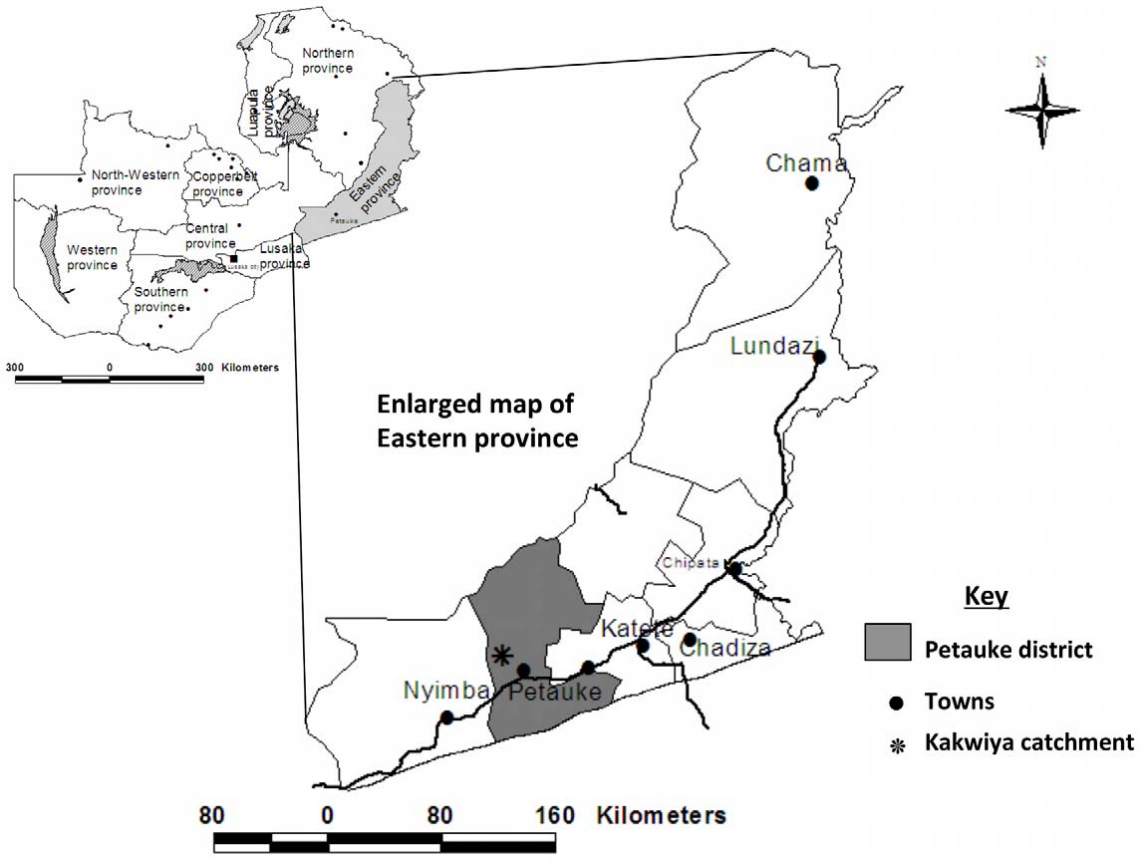
Map of Zambia indicating the study area (Kakwiya) in Petauke district of the Eastern province.

The study was conducted in a rural community (Kakwiya) in Petauke district of the Eastern province of Zambia (Figure 1). The community is serviced by a Rural Health Centre (RHC) whose catchment population is 11,344 (Clinic headcount records). The people in this community practice subsistence farming growing mostly crops like maize and groundnuts primarily for home consumption and cotton and bananas grown for household income generation. They also keep animals, mostly pigs with a few keeping cattle, goats and chickens. A preliminary visit to the area indicated that, as reported by Sikasunge *et al.* [21], there was a high number of free roaming pigs and reports of cysts observed in pigs slaughtered in backyards.

### Study design

A community-based cross sectional survey was conducted in the dry season between July and August 2009. The Kakwiya community was selected because it is a pig keeping community without any active ongoing sanitation programmes and cysticerci were observed in slaughtered pigs. The willingness of the community to participate in the study and the RHC to collaborate was also taken into account together with the availability of an adequate working space in the clinic and staff for the collection of samples. Only villages (n = 20) within a radius of 7 km from Kakwiya RHC were selected. The selected villages were visited and all persons invited to participate in the study. Each willing participant was, after written informed consent, given two plastic sample bottles and requested to submit a stool and a urine sample at the RHC. Upon submission of these samples, a blood sample was also taken by qualified health personnel. A questionnaire targeting household characteristics (such as number of inhabitants in a household, main household income, highest level of education attained, main source of drinking water), presence of household level risk factors (such as keeping of pigs and how they are kept, backyard slaughter of pigs, inspection of slaughtered pigs, consumption of pork by at least one member of the household, presence of a pit latrine, consumption or resell of clearly infected pork) and knowledge of the parasite (such as observation of tapeworm in human faeces, how people acquired a tapeworm, observation of cysts in pork meat, what the cysts were and how pigs acquired them) was administered to each participating household. At the same time geographic co-ordinates of each participating household were obtained using a Global Positioning System (GPS) receiver (eTrex Legend® Cx, Garmin).

### Sample collection and storage

Submitted stool samples were divided into two aliquots; one placed in 10% formalin and the other in 70% ethanol and these were stored at 4°C until use. Urine samples were aliquoted in 1.8 ml vials and stored at −20°C. Analyses and results for the urine specimens were discussed in our earlier report [22]. About 5 ml of blood were collected into sterile plain blood collecting tubes and allowed to clot. To obtain the maximum amount of serum, the blood tubes were allowed to stand at 4°C overnight and then centrifuged at 3000 *g* for 15 minutes. The supernatant (serum) was aliquoted into 1.8 ml vials and stored at −20°C until use. All the samples were transported to Lusaka where they were stored at −20°C until analysis.

### Coproscopic examination

Presence of helminth ova in stool was examined microscopically using the formalin-ether concentration technique [23]. Presence of a taeniid egg on a slide was recorded as being positive for taeniosis. The presence of other helminth eggs was also noted during the examination.

### Copro-antigen enzyme-linked-immunosorbent assay (Copro-Ag ELISA)

An in house copro-antigen detection ELISA (copro-Ag ELISA) as described by Allan *et al.* [24], with slight modifications, was performed on the stool samples. Briefly, the stool samples stored in 10% formalin were processed by mixing an equal amount of Phosphate Buffered Saline (PBS) and stool sample. This was allowed to soak for one hour with intermediate shaking and centrifuged at 2000 *g* for 30 minutes. The supernatant was then used in the ELISA. The assay involved coating polystyrene ELISA plates (Nunc® Maxisorp) with the capturing hyper immune rabbit anti-*Taenia* IgG polyclonal antibody diluted at 2.5 µg/ml in carbonate-bicarbonate buffer (0.06 M, pH 9.6). After coating, the plates were incubated for 1 hour at 37°C, washed once with PBS in 0.05% Tween 20 (PBS-T20) and all wells blocked by adding blocking buffer (PBS-T20+ 2% New Born Calf Serum). After incubating at 37°C for 1 hour and without washing, 100 µl of the stool supernatant was added and plates were incubated for 1 hour at 37°C followed by washing five times with PBS-T20. A biotinylated hyper immune rabbit IgG polyclonal antibody diluted at 2.5 µg/ml in blocking buffer was used as the detector antibody. One hundred microlitre was added and the plates were incubated for 1 hour at 37°C followed by washing 5 times. One hundred microlitre of Streptavidin-horseradish peroxidase (Jackson Immunoresearch Lab, Inc.) diluted at 1/10,000 in blocking buffer was added as conjugate. After 1 hour incubation at 37°C and washing 5 times, 100 µl of ortho phenylenediamine (OPD) substrate, prepared by dissolving one tablet in 6 ml of distilled water and adding 2.5 µl of hydrogen peroxide, was added. The plates were incubated in the dark for 15 minutes at room temperature before stopping the reaction by adding 50 µl of sulphuric acid (4 N) to each well. The plates were read using an automated spectrophotometer at 490 nm with a reference of 655 nm. To determine the test result, the optical density (OD) of each stool sample was compared with the mean of a series of 8 reference *Taenia* negative stool samples plus 3 standard deviations (cutoff).

### Serum antigen enzyme-linked-immunosorbent assay (sero-Ag ELISA)

Presence of circulating cysticercus antigens was measured by the monoclonal antibody based B158/B60 Ag-ELISA (sero-Ag ELISA) [25], [26]. Sera from two known highly positive pigs (obtained from a local pig market and confirmed by dissection) were used as positive controls. The OD of each serum sample was compared with a sample of negative serum samples (N = 8) at a probability level of P = 0.001 to determine the result in the test [26].

### Differentiation of *Taenia* spp

Differentiation of the *Taenia* spp. was done using molecular methods. Taeniosis positive individuals were treated with niclosamide (2 g single dose) followed by a purgative (Magnesium sulphate, 30 g). The collected tapeworm segments were stored in 70% ethanol until use. DNA was extracted from the parasitic material using the Boom extraction method slightly modified as described by Rodriguez-Hidalgo *et al.* [27] and PCR used to amplify the mitochondrial 12 s rDNA gene fragment. Restriction fragment length polymorphism (RFLP) was then used to differentiate the *Taenia* spp. [27].

### Statistical analysis

All collected data was entered into an Excel (Microsoft Office Excel 2007®) spreadsheet and analyses were conducted in Stata 10 (http://www.stata.com). Chi square test was used to check for differences between disease positivity and gender. Uni- and multivariate logistic regressions were used to investigate the relationship between taeniosis and cysticercosis positivity and individual gender and age. The age variable was first used as a continuous variable and then categorized into 10 categories of 10 years each, in order to identify changes in positivity frequencies as a function of the age of individuals. A change point analysis was used to simplify the observed relations into antigen patterns as a function of age [28], [29]. The level of significance was set at *p*<0.05 for all statistical analyses.

The geo-reference data collected was used for spatial analysis using ArcView Gis 3.2 (http://www.esri.com). Analysis of the possibility of geographical clustering of households with latrines or those that kept pigs and also cases of taeniosis and cysticercosis was done by means of the risk-adjusted nearest neighbour hierarchical spatial clustering (Rnnh) using Crimestat® III [30]. Given the limited number of individuals infected with taeniosis and cysticercosis, the minimum number of cases per cluster was set at 3 while the minimum number of households with a latrine or that kept pigs was set at 20. Monte Carlo simulations were run in this software to determine the significance of the clusters. Significance level of a cluster in the simulation was set at 95% and a cluster was determined significant if the density of the points was higher than that obtained at the 95^th^ percentile after 1000 simulations.

## Results

### Study population

**Figure 2:**
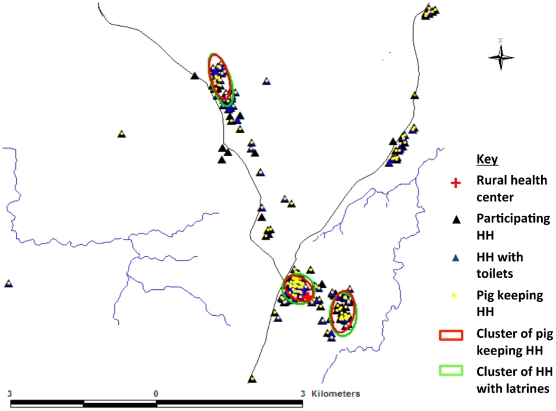
Spatial pattern of the participating households. Map of the study community showing participating households (HH), HH with pit latrines and pig keeping HH.

A total of 720 willing participants from 20 villages belonging to 255 households participated in the study. Of these, 428 (59.4%) were females and 292 (40.6%) were males and the age ranged from 1 to 96 years with a median age of 12 years. The age distribution, with a majority of the younger age group, was typical of rural areas in developing countries [31]. The number of people living in a household ranged from 1 to 13 with a median of 7. Seven hundred and eighteen of the participants gave a stool sample and 708 gave a blood sample. At least one participant from each participating household gave a sample depending on the willingness of the household members. The number of individuals sampled from each household ranged from 1 to 11. Some household characteristics recorded from the questionnaire administered to the 255 households included; 32.6% kept pigs with 99.6% of these rearing on free-range, 47.8% of the households did not have latrines (Figure 2) and 94.5% of the households had at least one individual who consumed pork. Three clusters each of households with latrines (14.13881S, 31.19501E, density = 748.97; 14.14338S, 31.20369E, density = 151.95 and 14.09718S, 31.17940E, density = 117.15; 95^th^ percentile density = 0.02) and those that kept pigs (14.13891S, 31.19493N, density = 299.89; 14.14390S, 31.20374E, density = 134.33 and 14.09773S, 31.17961E, density = 86.79; 95^th^ percentile density = 0.01) were identified in the study area (Figure 2). About 44% of the households reported to have slaughtered a pig in their backyards. None of them had the meat inspected before either home consumption or resell to members of the community. Pork was reported to be consumed in a variety of ways including boiling, frying and roasting. The data obtained in the questionnaire on risk factors is described in more detail in another report (Mwape *et al.*, submitted article).

### Prevalence of taeniosis

**Figure 3:**
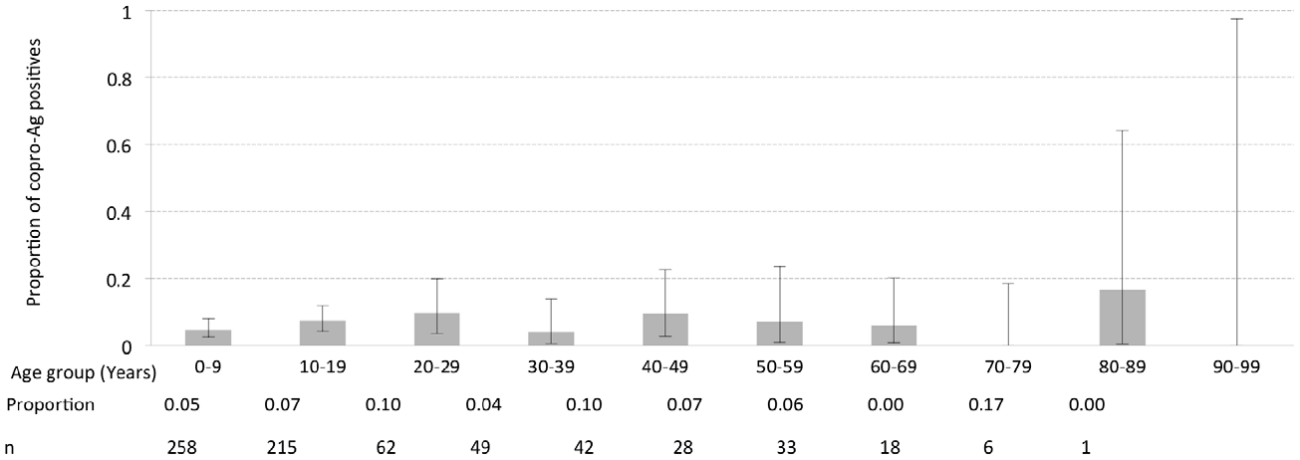
Taeniosis prevalence per age group. Results of the copro-Ag ELISA in a function of the 10 age groups of 10 years each. The upper and lower 95% exact binomial confidence intervals for the prevalence in each age group are represented through error bars. The proportion of positives and number of individuals sampled in each age group are also shown.

**Figure 4:**
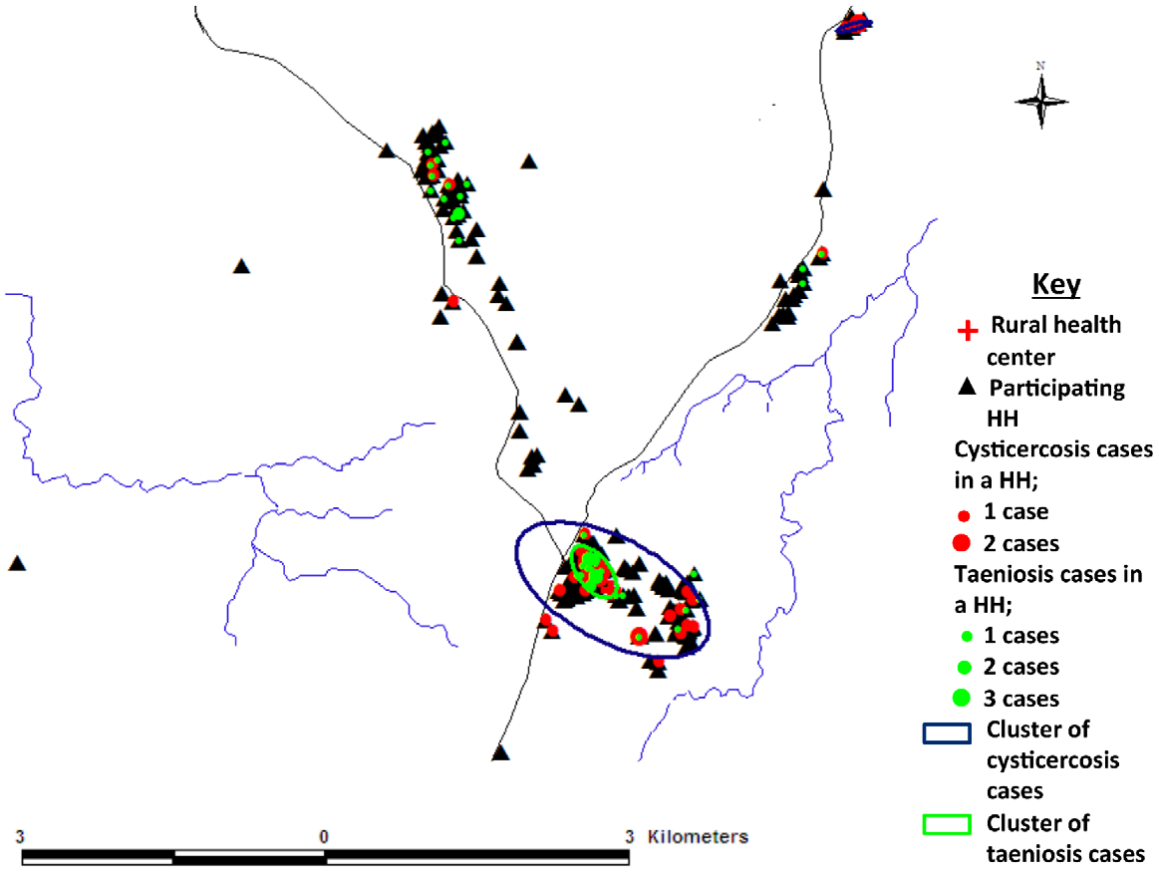
Spatial pattern of diagnosed taeniosis and cysticercosis cases. Map of the study community showing cysticercosis and taeniosis cases by sero-Ag ELISA and copro-Ag ELISA at household level; respectively.

The results for both the coproscopic examination and copro-Ag ELISA are shown in Table 1. Two (0.3%) individuals were positive for taeniosis on coproscopic examination while copro-Ag ELISA detected 45 (6.3%) positives. The two coproscopic positives were also positive on copro-Ag ELISA. Figure 3 shows the copro-Ag ELISA results in function of 10 age groups of 10 years each. The highest prevalence was determined in the 80–89 years age group, though this was not significantly different from the other age groups. A univariate logistic regression analysis did not indicate any relationship between copro-Ag ELISA positivity and sex (*p* = 0.548) or age (*p* = 0.311). This finding was the same for the multivariate analysis with *p* values of 0.139 and 0.645 for sex and age respectively. One cluster of taeniosis cases (14.13868S, 31.19509E, density 116.55; 95^th^ percentile density = 49.33) was identified in the study community (Figure 4). At household level, the number of positives per household ranged from 0 to 3. All taeniosis positive individuals were treated with 2 g niclosamide *per os* and given a purgative (Magnesium sulphate) two hours later. One tapeworm was collected and confirmed to be *T. solium* by PCR-RFLP.

**Table 1:** Results of the coproscopic examination and copro-Ag ELISA of the stool samples.

Diagnostic test	Number of positive individuals	Prevalence % (95% CI)
Coproscopic examination	2/718	0.3 (−0.1–0.7)
Copro-Ag ELISA	45/712*	6.3 (4.5–8.1)

*: No sample was left in 6 containers after coproscopic examination for copro-Ag ELISA as very little material was provided by the participants.

Results of the coproscopic examination and copro-Ag ELISA of the stool samples from Kakwiya Rural Health Centre together with their 95% confidence intervals.

### Prevalence of cysticercosis

**Figure 5:**
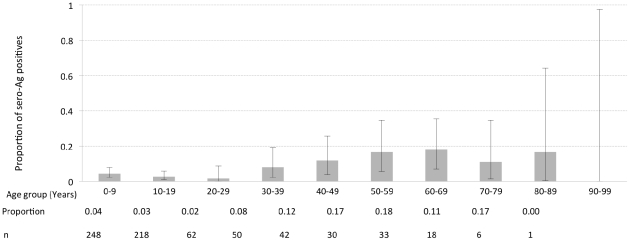
Cysticercosis prevalence per age group. Results of the sero-Ag ELISA in a function of the 10 age groups of 10 years each. The upper and lower 95% exact binomial confidence intervals for the proportion in each age group are represented through error bars. The proportion of positives and number of individuals sampled in each age group are also shown.

**Figure 6:**
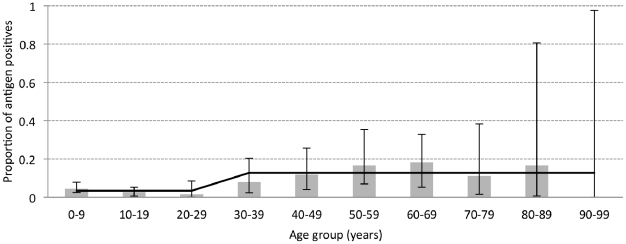
Change point analysis. Results of the sero-Ag ELISA (grey bars) in a function of 10 age groups of 10 years each, including the logistic regression predictions (black line). The upper and lower 95% exact binomial confidence intervals are shown with error bars.

The results for the sero-Ag ELISA are shown in Figure 5 in function of 10 age groups of 10 years each. Circulating cysticercus antigens were detected in 41 (n = 708) participants giving an apparent prevalence of 5.8% (95% CI, 4.1–7.5). Uni- and multivariate logistic regression analysis revealed a very strong relationship between sero-Ag positivity and age (*p*<0.001). Figure 6 indicates that the prevalence of cysticercosis is initially low and a change point analysis indicated a significant increase in positivity frequencies at 30 years of age. The logistic regression model indicated that the proportion of sero-Ag ELISA positive individuals remains at a constant level until the age of 30, and from this age onwards a significantly higher level is observed (*p*<0.001).

A relationship was observed between copro-Ag positivity and sero-Ag positivity (*p* = 0.03) indicating that a copro-Ag positive individual was at an almost three-fold higher risk of being sero-Ag positive than the one who was not (OR = 2.9, *p* = 0.029).

There was no statistically significant difference in prevalence between males and females (χ^2^ = 0.034, *p* = 0.854). Two clusters of cysticercosis cases (14.14048S, 31.19692E, density 31.31; 14.08460S, 31.22085E, density 195.17; 95^th^ percentile density = 23.16) were identified in the study community with the larger cluster spatially related to the taeniosis cluster (Figure 4).

### Prevalence of other parasites

Other intestinal parasites detected on coproscopic examination included hookworms, *Schistosoma mansoni*, *Trichuris trichiuria* and *Ascaris lumbricoides*. Table 2 shows the prevalence rates for these parasites with their respective 95% confidence intervals.

**Table 2:** Prevalence rates of other parasites diagnosed on coproscopic examination of the stool samples.

Parasite	Number positive	Prevalence % (95% CI)
Hookworms	107	14.9 (12.3–17.5)
*Trichuris trichiuria*	69	9.6 (7.4–11.8)
*Schistosoma mansoni*	16	2.2 (1.1–3-3)
*Ascaris lumbricoides*	6	0.8 (0.2–1.5)

Prevalence rates of other parasites diagnosed on coproscopic examination of the stool samples and their 95% confidence intervals (n = 718).

## Discussion

The objective of this study was to determine the prevalence of taeniosis and cysticercosis in a rural community in the Eastern Province of Zambia, where risk factors for the transmission of *T. solium* are present.

### Taeniosis

*T. solium* taeniosis tends to have a low prevalence, typically less than 1%, even in endemic communities [32], a higher prevalence is considered hyper-endemic [33]. In this study a prevalence of 6.3%, based on copro-Ag ELISA, was determined, indicating a hyper-endemicity in the study community. As in a number of other studies, no significant association between age/sex and taeniosis positivity could be determined [31]–[35].

Even though similar high taeniosis prevalences have been recorded in Kenya (4–10%) [17], the 6.3% prevalence determined in this study should be looked at critically. The sensitivity and specificity of the copro-Ag ELISA are estimated at 96–98% and 98–100%; respectively [5], [36]. However, the possibility of false positive test results due to cross-reactions with other pathogens present in the community should be considered. The assay has been reported not to cross-react with other parasite species including *A. lumbricoides*, *T. trichiuria*, *Hymenolepis nana*, *H. diminuta* or parasitic protozoa [36]. Also in our laboratory, stool samples with known *H. nana*, *Schistosoma* spp., *T. trichiuria* and *A. lumbricoides* infections were analyzed, and all results remained under the cut off level (Unpublished data). As the assay is not species specific [24], the possibility of the high taeniosis prevalence to be partially due to *T. saginata* infections cannot be ruled out. However, bovine cysticercosis in Zambia has so far only been reported in the Central and Southern provinces [37] and Western province (I.K. Phiri, personal communication).

Interviews with local people in the study area revealed that pig slaughter and pork consumption increases in the dry season as it is time for harvest and residents have then the means to buy either an entire pig or pieces of pork for home consumption. During this period, pig owners not only slaughter more pigs for the market but also for their own home consumption. Higher pork consumption could have led to new (still immature) taeniosis infections at the time of sampling, which will be detected by copro-Ag ELISA but not yet by coprology [5].

Only one tapeworm (from a participant positive on both copro-Ag and coproscopic examination) could be recovered after treatment of the 45 copro-Ag positive participants. The low recovery rate of tapeworms can be explained by: (1) stools were obtained only over one day and not over 3 days post treatment [38] due to logistical constraints, (2) usually after antiparasitic treatment, small and unrecognisable fragments are expelled by most patients [38] and these are easily missed, and (3) treatment of copro-Ag positive individuals was conducted over six months after collection of stool samples; natural expulsion may have occurred in this period.

### Cysticercosis

The sero-Ag ELISA assay detected an apparent cysticercosis prevalence of 5.8% indicating the presence of viable cysts and as such active infections in these individuals. The prevalence of cysticercosis recorded in our study is comparable with that recorded in other endemic areas such as in the Andean region of Ecuador and in north Vietnam [39], [40], higher than in west Cameroon (0.4 to 4.0%) and southern Ecuador (2.3%) [41], [42] but lower than that reported in the Democratic Republic of Congo (21.6%) [14]. Other studies that have recorded higher seroprevalences include those that used antibody detection techniques such as in Mozambique (12.1%), South Africa (7.4%) and Peru (13.9%) [15], [16], [31]. However, antibody detection indicates exposure to the parasite and not necessarily established infection and hence is likely to detect more positives than the antigen detecting assay used in the current study [43].

Change point analysis of the association of antigen seropositivity and age revealed that the number of individuals in which circulating antigens were detected was significantly higher in people older than 30 years, indicating that viable cysts were more frequently present in individuals above this age. Studies have shown that a higher proportion of vesicular stage cysticercii is found in older (60 years and above) NCC patients [44], [45] and this has been attributed to immunosenescence since a weaker immunity in the elderly would facilitate the establishment and maintenance of viable cysticercii unlike in fully immunocompetent younger individuals [46]. The significant increase in sero-antigen positive individuals in the elderly was also observed in Ecuador where the number of positive individuals was higher in people order than 60 years [28]. However, in our study we see an increase already in young people (from 30 years onwards) who are supposed to be immunocompetent.

Establishment and development of infection is influenced by a range of complex factors; among which are parasite factors (e.g. parasite virulence/pathogenicity influenced by genetic differences, number, stage, location), host factors (e.g. age, gender, genetics influencing the immunological responses of the host when exposed to infection) and environmental factors (e.g. presence of risk factors, level of exposure, presence of other infections) [47]. It is very difficult to pinpoint exactly those factors present in the study area/population/age groups; that can explain this early increase in establishment of viable infection.

The high taeniosis prevalence recorded in the study community entails a possible very high exposure risk to infective eggs. In a study in India, higher infection rates (as indicated by sero Ag detection) were noted in areas with higher taeniosis prevalences [48]. Also in our study a significant positive relationship between copro-Ag positivity (presence of tapeworm) and sero-Ag positivity (cysticercosis) was established (logistic regression and cluster analysis). Level of exposure/infection with which the host is confronted can have an important effect on the immunological response of the host [44], and as such on the establishment of viable infection.

General factors that lower immunity in groups of individuals in the population could be at play making them more susceptible to infection. According to the United Nations Human Development Indices of 2008, about 64% of Zambia's population lived on less than $1 per day as compared to only 20% for Ecuador [49]. Poverty is an indication for poor nutritional status, which has an impact on the immune system [50]. Also the presence of other diseases such as HIV-AIDS, malaria and tuberculosis, other helminthic infections and physical environmental conditions [51] can influence greatly the host's reaction to other infections. In 2008, Zambia's HIV prevalence stood at 14.3% with the age group between 20 and 40 years being the most affected [52]. The country is also endemic for malaria [53] and helminthic infections are widely reported in rural areas, as reported in this study.

Genetic polymorphism of the parasite is another important determining factor for the establishment and development of infection. Nakao *et al.* [54] have described a cluster of isolates from Asia, and another cluster from isolates from Latin America and Africa. However, genetic differences within a cluster (within a continent/country/region) need to be evaluated as well. Several Zambian isolates are currently being examined, and preliminary results indicate a high genetic variability (Unpublished results, K. Kanobana), which might explain differences in development of infection between regions.

We have, in this study, shown that *T. solium* taeniosis and cysticercosis are present in the study community. Many issues remain unclear and obviously more work is required to understand the many factors that contribute to the transmission dynamics of the parasite and disease development in endemic rural areas. Also the economic impact and burden of disease in rural pig keeping communities of Zambia needs to be determined.

## Supporting Information

Checklist S1 STROBE checklist
(DOC)
